# A Pilot Randomized Crossover Trial of Wet Suction and Conventional Techniques of Endoscopic Ultrasound-Guided Fine-Needle Aspiration for Upper Gastrointestinal Subepithelial Lesions

**DOI:** 10.1155/2021/4913107

**Published:** 2021-03-22

**Authors:** Mika Takasumi, Takuto Hikichi, Minami Hashimoto, Jun Nakamura, Tsunetaka Kato, Hitomi Kikuchi, Yuichi Waragai, Ko Watanabe, Tadayuki Takagi, Rei Suzuki, Mitsuru Sugimoto, Manabu Hayashi, Yuki Sato, Hiroki Irie, Ryoichiro Kobashi, Yoshinori Okubo, Masao Kobayakawa, Hiromasa Ohira

**Affiliations:** ^1^Department of Gastroenterology, Fukushima Medical University School of Medicine, Fukushima, Japan; ^2^Department of Endoscopy, Fukushima Medical University Hospital, Fukushima, Japan; ^3^Department of Medical Research Center, Fukushima Medical University, Fukushima, Japan

## Abstract

**Methods:**

Twenty-six patients with UGI-SELs indicated for EUS-FNA were randomly assigned to the dry-first arm using the dry technique for the first two passes or the wet-first arm using the wet technique for the first two passes using a cross-over design with a ratio of 1 : 1. The primary endpoint was the cellularity score of the EUS-FNA specimens rated on a 4-point scale (0-3). The secondary endpoints were the factors influencing cellularity in each suction technique.

**Results:**

The mean cellularity score was 1.65 ± 1.20 for the wet technique and 2.00 ± 0.98 for the dry technique (*p* = 0.068). Logistic regression analysis showed that higher cellularity may be related to the final diagnosis of gastrointestinal stromal tumors in the dry technique and the SEL location in the upper stomach in the wet technique.

**Conclusion:**

The wet EUS-FNA technique failed to show a potential for improved cellularity of specimens compared to the dry technique for UGI-SELs.

## 1. Introduction

Endoscopic ultrasound- (EUS-) guided fine-needle aspiration (EUS-FNA) is a commonly used method to obtain specimens from gastrointestinal (GI) subepithelial lesions (SELs) [1]. The diagnostic accuracy of EUS-FNA for upper GI SELs (UGI-SELs) was reported to be 62.0-93.4% [1–5]; however, it is lower than that of lymph node or extraluminal masses [6]. In a recent study, the wet suction technique (“wet” technique) has been reported to be a novel way to enhance the quality of EUS-FNA specimens [7].

The wet technique involves flushing the needle with saline to replace the column of air within the lumen of the needle before needle aspiration, while a conventional EUS-FNA technique (so-called “dry suction technique; we defined this as “dry” technique in this article) applies negative pressure suction on an empty needle lumen after the stylet is removed. Several papers have reported on a wet EUS-FNA technique for various solid lesions such as mediastinal, pancreatic, nonpancreatic intra-abdominal, or pelvic cavity masses [7–10] and for liver biopsy [11, 12] with improved tissue adequacy compared to the dry technique. However, no studies have reported on the use of the wet technique for UGI-SELs, for which it can be difficult to obtain specimens compared to lymph node and extraluminal masses.

We hypothesized that the tissue adequacy of the “wet” technique is superior to that of the dry technique. Therefore, we conducted a prospective pilot comparison in order to explore whether the wet technique would present better outcomes in the specimen quality of UGI-SELs compared to the dry technique.

## 2. Patients and Methods

### 2.1. Study Design

This study was a randomized crossover trial conducted at Fukushima Medical University Hospital. Patients with UGI-SELs indicated for EUS-FNA were randomly assigned to the dry-first arm using the dry technique for the first two passes, or the wet-first arm using the wet technique for the first two passes in a 1 : 1 ratio. Following a cross-over design, the pass sequence for the dry-first arm was dry, dry, wet, and wet. For the wet-first arm, the pass sequence was wet, wet, dry, and dry. Randomization was performed by sequentially opening numbered opaque envelopes containing computer-generated group allocation cards in a random sequence. The operators, assistants, and pathologists were not blinded.

This study was conducted according to the Declaration of Helsinki, approved by the Ethics Committee of Fukushima Medical University (approval No. 2207), and registered in the University Hospital Medical Information Network (as UMIN 000017031).

### 2.2. Patient Acquisition

**Figure 1:**
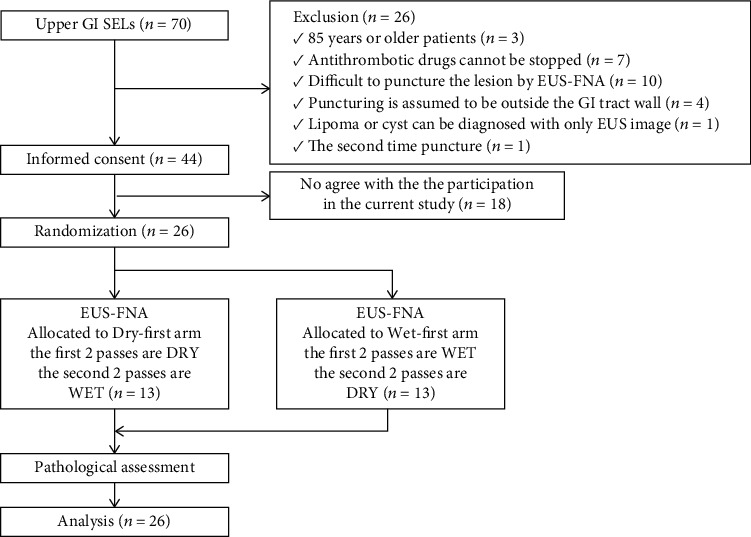
Flowchart illustrating the randomization and recruitment process of the study. GI: gastrointestinal; SEL: subepithelial lesion; EUS-FNA: endoscopic ultrasound-guided fine-needle aspiration; DRY: dry technique; WET: wet technique.

Patients ≥ 18 years of age referred for EUS-FNA of UGI-SELs from April 2015 to July 2019 were offered the opportunity to participate in the study. The inclusion criteria were a SEL of the esophagus or stomach that had a tumor size of ≥10 mm by EUS that could be punctured with EUS-FNA. The exclusion criteria were as follows: (1) patients aged ≥85 years, (2) use of antithrombotic drugs that were not able to be discontinued, (3) lesions that would be difficult to puncture, (4) presence of a thick blood vessel in the puncture line, (5) lesions assumed to be outside the GI tract wall, (6) a lipoma or cyst that could be diagnosed by EUS imaging, and (7) previous history of puncture of the target lesions before study entry. Participants were randomly allocated into one of two arms as described above (the dry-first or wet-first arms) (Figure 1).

### 2.3. EUS-FNA

**Figure 2:**
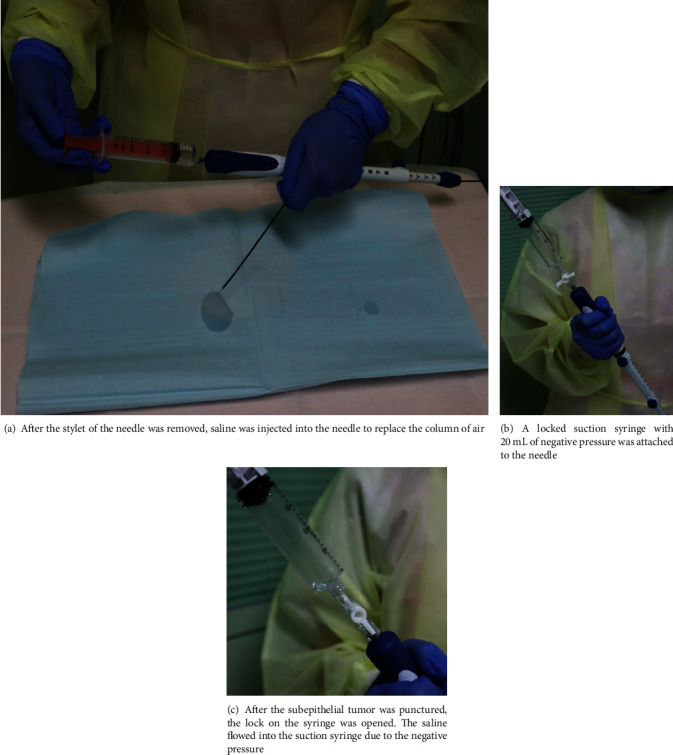
Wet technique of endoscopic ultrasonography-guided fine-needle aspiration.

The EUS-FNAs without rapid on-site evaluation [13] were performed with a linear or convex array EUS gastrovideoscope (GF-UCT260 or TGF-UC260J; Olympus Corp., Tokyo, Japan) using a 22-gauge needle (Expect™; Boston Scientific Corp., Marlborough, MA, USA). The wet technique was performed as previously reported [7, 10]. Briefly, a stylet was removed from an EUS-FNA needle, then the needle was flushed with 5 mL of saline to replace the column of air (Figure 2(a)), and a 20 mL syringe was attached in a “locked” position to the needle (Figure 2(b)). Once the SEL was identified and the intervening blood vessels were excluded, the needle was punctured into the SEL. Subsequently, suction was applied at 20 mL (Figure 2(c)), and the needle moved back and forth within the SEL about 20 times to collect the sample. Patients received deep sedation and monitored anesthesia care throughout the procedures. For the dry technique, the SEL was punctured by the needle with the stylet and then negative pressure suction was applied to the empty needle after the stylet was removed. After that, the dry technique follows the same steps as the wet technique. All procedures were performed by endoscopists with at least 5-year-EUS-FNA experiences under the supervision of an experienced endoscopist with over 15 years of EUS-FNA experiences.

### 2.4. Outcomes

The primary endpoint was the level of cellularity in the EUS-FNA specimens. Secondary endpoint was the factor influencing the cellularity in each suction technique

**Figure 3:**
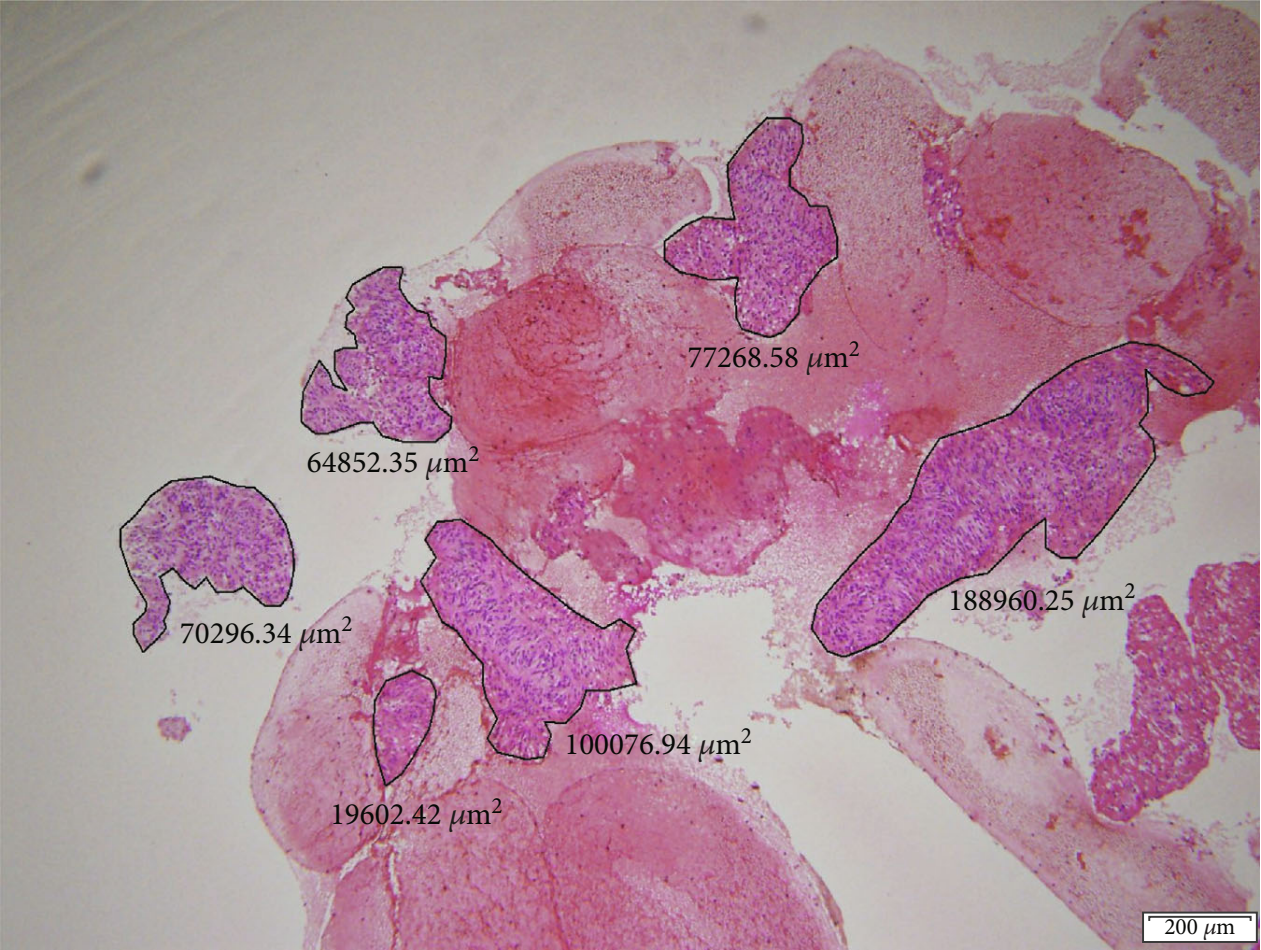
Measurement of cellularity area in the cell-block specimens. The specimens which were obtained by two punctures of each technique were combined into one. The area was then measured at the point of the maximum cross section using a soft imaging microscope.

The specimens, which were obtained by two punctures of each technique, were combined into one. In other words, specimens obtained with the same technique of the same arm were consolidated into one. The area of cellularity in the cell-block specimens was then measured at the maximum cross section using a soft imaging microscope (cellSens Standard 1.11; Olympus Corp.) and evaluated by a medical doctor who did not participate in the study and was blinded (Figure 3). The cellularity score was rated using a 4-point scale as follows: 0 = no cellularity, 1 = sparse cellularity (0–10,000 *μ*m^2^), 2 = moderate cellularity (10,000–100,000 *μ*m^2^), and 3 = high cellularity (>100,000 *μ*m^2^). According to Attam et al., the score was also divided into two categories as follows: acellular and poor cellularity (score 0 and 1) and moderate and high cellularity (scores 2 and 3) [7].

The factors evaluated for the association with the cellularity categories were as follows: first-pass technique (dry-first arm vs. wet-first arm), tumor size (<20 mm vs. ≥20 mm), SEL location (others vs. upper stomach), and final diagnosis (gastrointestinal stromal tumor (GIST) vs. others, including cases that were not diagnosed by EUS-FNA). The final diagnosis was determined based on the EUS-FNA specimens. All patients diagnosed with GIST by EUS-FNA underwent surgery and were finally diagnosed using surgical specimens.

### 2.5. Sample Size Calculation and Statistical Analysis

Based on a review of the literature regarding suction techniques for heterogeneous indications [7], we expected that the mean cellularity scores for the dry and wet techniques would be 1.45 ± 0.76 and 1.82 ± 0.76, respectively, and the correlation coefficient would be 0.8. Our calculations yielded target sample sizes of 22, with a power of 0.8 and an *α* value of 0.1 using the statistical software EZR (version 1.27; Saitama Medical Center, Jichi Medical University, Saitama, Japan) [14]. Assuming a 20% dropout or withdrawal rate, we calculated a final sample size of 26 patients (13 per arm). IBM SPSS Statistics software (version 21; IBM Corp., Armonk, NY, USA) was used for the statistical analysis. Differences between groups were compared using a Wilcoxon signed-rank test, chi-squared test, and Fisher's exact test. In those analyses, a two-tailed distribution was used. Factors with *p* values < 0.20 in the univariate analyses were included in a multivariate logistic regression analysis to assess the significant predictors of obtaining sufficient specimens (acellular and poor cellularity versus moderate and high cellularity). Statistically significant differences were defined as those having *p* values < 0.1 for the primary endpoint and <0.05 for the secondary endpoints.

## 3. Results

### 3.1. Patient Characteristics and SEL Features

A total of 26 patients with UGI-SELs were enrolled in the study. All participants were assessed by EUS-FNA and included in the final analysis. Patient characteristics are shown in Table 1. The median age was 68 years (range, 19–83 years), and 38% of the patients were women. The median size of the SELs was 23 mm (range, 13–87 mm). The accuracy of EUS-FNA was 92.3% (12/13) in the wet-first arm and 84.6% (11/13) in the dry-first arm. The features of the SELs, including their locations and sizes, are also summarized in Table 1. No patient experienced adverse events from EUS-FNA. In addition, among the three cases in which the diagnosis was not confirmed by EUS-FNA, one case was diagnosed as schwannoma as a result of surgical resection. The other two cases are under follow-up, but no change has been observed.

#### 3.1.1. Cellularity

For both techniques, all patients underwent EUS-FNA as per the protocol. The mean cellularity score was 1.65 ± 1.20 and 2.00 ± 0.98 for the wet and dry techniques, respectively (*p* = 0.068). The *p* value of the primary endpoint exceeded the prespecified significance level of 0.1. The proportion of moderate and high cellularity specimens was significantly higher with the dry technique than with the wet technique (77.0% vs. 61.5%; *p* = 0.018; Table 2). Regarding the first-pass technique (study arm), there were no differences in the mean cellularity scores between the specimens obtained by the wet and dry techniques in the dry-first arm; however, the wet technique yielded lower mean cellularity scores when used as the first-pass technique (*p* = 0.031; Table 3).

### 3.2. Factors Influencing Cellularity

Logistic regression analysis showed that the SEL location of the upper stomach was an independent factor associated with moderate and high cellularity with the wet technique (adjusted odds ratio: 0.125; 95% confidence interval: 0.018–0.858; *p* = 0.034). Furthermore, a final diagnosis of GIST was an independent factor associated with moderate and high cellularity with the dry technique (adjusted odds ratio: 9.079; 95% confidence interval: 1.012–81.485; *p* = 0.049). Other factors such as the first-pass technique (study arm) and size of the SEL were not significant (Table 4).

## 4. Discussion

This study is the first prospective comparison of the wet and dry techniques of EUS-FNA for UGI-SELs. Based on the results of previous studies [7–12], in which the wet technique of EUS-FNA resulted in a better total volume of aspirate and better specimen adequacy, we hypothesized that the tissue adequacy of the wet technique would also be superior to the dry technique for UGI-SELs. However, the superiority of the wet technique over the dry technique could not be demonstrated for UGI-SELs. The results were contrary to our expectations. Furthermore, in the wet-first arm, even if the wet technique was used as the first-pass technique, the mean cellularity result was insufficient than that of the dry technique as a second pass.

In this study, there were two reasons why the wet technique did not show superiority in EUS-FNA of UGI-SELs. One reason was that the lesion moved with the gastric wall during the puncture of SELs, and the needle could not be moved smoothly inside the lesion, which prevented us from taking advantage of the WET method, which is the maintenance of suction pressure. Another reason may be the stiffness of the SELs. Of the 26 patients in this study, 25 had gastric SELs, of which the most common was GIST, followed by leiomyoma. GISTs and other mesenchymal tumors are stiffer than pancreatic tumors and enlarged lymph nodes, for which the superiority of the WET method over the DRY method has been demonstrated. GISTs and schwannomas have been proven to have high stiffness on EUS-elastography as assessed by UGI-SELs [15, 16]. In fact, in this study, one of the three cases that could not be diagnosed due to the small amount of specimens collected was a schwannoma, which was confirmed by surgically resected specimen. In EUS-elastography, leiomyoma is also reported to be harder than ectopic pancreas, although not so hard as GIST or schwannoma [16]. Due to the hardness of SEL, even if puncture is possible, it may be difficult to obtain inadequate specimens using only the force of aspiration, which is considered to be the reason why the advantages of the WET method could not be properly utilized. In addition, the inadequate overall diagnostic accuracy of 88% in the present study may have influenced the failure to demonstrate the significance of the WET method. In an analysis of factors associated with the diagnostic accuracy of EUS-FNA for gastrointestinal SEL [17], it has been reported that concomitant use of ROSE and more than three punctures improve it.

We then evaluated the factors for sampling adequacy in each method. GIST as the EUS-FNA diagnosis with the dry technique and the upper stomach as the location with the wet technique were found to be significant factors to obtain the adequate sample in the multivariate analysis. Previous reports have also shown that the diagnostic accuracy of EUS-FNA for GIST is higher than that for leiomyoma and schwannoma [18, 19]. Compared to leiomyoma and schwannoma, GISTs often have heterogeneous internal echogenicity on EUS. This indicates that the internal histology and cell arrangement of GISTs are not homogeneous. This suggests that the specimen adequacy by aspiration is higher in GISTs than in leiomyoma or schwannoma because of the possibility of lower cell-cell connectivity. However, compared with pancreatic tumors and lymph nodes, the specimen adequacy is still low. Next, the reason why the sampling adequacy of the upper stomach was higher than that of the other sites may be attributed to the fact that the EUS scope can easily be placed close to the lesion, which facilitates subsequent puncture. In addition, when the needle is moved in the lesion located in the upper stomach after puncture, the gastric wall can be avoided from escaping. In the case of the middle stomach or lower stomach, it may be difficult to approach the lesion, and the gastric wall may be affected by movement during puncture, making it impossible to obtain samples in the SEL even if puncture can be performed.

The advantage of the wet technique is that a needle filled with water aspirates the tissue for a much longer distance than a needle filled with air [10], but for a hard tumor like a SEL, an adequate tissue specimen could not be obtained with only uniform suction pressure. Therefore, the results of this study suggest that another method should be chosen instead of the aspiration method in order to obtain sufficient tissue under EUS-guidance for UGI-SELs. Recently, a method of EUS-guided tissue acquisitions by using a needle capable of cutting out tissue (Procore™, Acquire™, Sharkcore™, etc.) has been termed EUS-guided fine-needle biopsy (EUS-FNB) [20–25]. In a comparative study of EUS-FNA and EUS-FNB for pancreatic lesions, the diagnostic accuracy was the same for both methods at 83.3%, but fewer punctures were reported for EUS-FNB (FNB vs. FNA, 1.11 vs. 1.83, respectively; *p* < 0.05) [20]. In addition, EUS-FNB was superior in the evaluation of sample quality by pathologists [20]. In a comparative study of 19G FNA and 22G FNB needles, which also included gastric SELs, the 22G FNB needle had a better sampling adequacy (FNB vs. FNA, 67.4% vs. 94.1%, respectively; *p* = 0.032) [21]. In a comparative study between EUS-FNA and EUS-FNB, which included other gastric SELs, a higher tissue sampling volume was reported for EUS-FNB [22, 23]. Even in a study that focused on SELs, EUS-FNB had higher diagnostic accuracy than EUS-FNA (FNB vs. FNA, 88.03% vs. 77.19%, respectively; *p* = 0.030) and fewer punctures (FNB vs. FNA, 2.94 vs. 3. 55, respectively; *p* = 0.003) have been reported [24]. As a technique without EUS-guidance, mucosal incision biopsy has been reported [17, 26]. However, although this method has the same diagnostic accuracy as EUS-FNA, it is more invasive to the patient and the procedure is more complicated.

This study has several limitations. First, this study was conducted at a single institution. Second, several unexperienced endoscopists performed the EUS-FNAs, though all EUS-FNAs were performed under supervision by an experienced endoscopist. Third, the pathologists evaluating EUS-FNA specimens were not blinded. Finally, the FNB needle was not commercially available at the beginning of this study.

## 5. Conclusion

The wet technique of EUS-FNA was not more effective than the dry technique for UGI-SELs. The higher cellularity was related to the final diagnosis of GIST in the dry technique and the SET location in the upper stomach in the wet technique.

## Figures and Tables

**Table 1:** Patient characteristics (*n* = 26).

Sex^∗^	Male	16 (61.5)
	Female	10 (38.5)
Age^∗^ (years)	<70	15 (57.7)
	≧70	11 (42.3)
Location^∗^	Esophagus	1 (3.8)
	Upper stomach	15 (57.7)
	Middle stomach	7 (27.0)
	Lower stomach	3 (11.5)
Size^∗^ (mm)	<20	15 (57.7)
	≧20	11 (42.3)
Final diagnosis of EUS-FNA^∗^	GIST	18 (69.2)
	Leiomyoma	3 (11.5)
	Cancer	1 (3.8)
	Aberrant pancreas	1 (3.8)
	Unknown	3 (11.5)
Diagnostic accuracy	Dry-first arm	11/13 (84.6)
	Wet-first arm	12/13 (92.3)

EUS-FNA: endoscopic ultrasound-guided fine-needle aspiration; GIST: gastrointestinal stromal tumor. ^∗^*n* (%).

**Table 2:** Quantity of cellularity in the EUS-FNA specimens.

	DRY (*n* = 26)	WET (*n* = 26)	*p* value
First pass, number (%)	13 (50.0)	13 (50.0)	
Specimen cellularity score, number (%)			
0	3 (11.5)	7 (26.9)	
1	3 (11.5)	3 (11.5)	
2	11 (42.3)	8 (30.8)	
3	9 (34.6)	8 (30.8)	
Cellularity score, mean ± SD	2.00 ± 0.98	1.65 ± 1.20	0.068
Moderate and high cellularity^∗^, *n* (%)	20 (77.0)	16 (61.5)	0.018
Acellular and poor cellularity^∗∗^, *n* (%)	6 (23.0)	10 (38.5)	

EUS-FNA: endoscopic ultrasound-guided fine-needle aspiration; DRY: dry technique; WET: wet technique; SD: standard deviation. ^∗^Specimen cellularity scores of 2 and 3. ^∗∗^Specimen cellularity scores of 0 and 1.

**Table 3:** Specimen cellularity of each technique in each arm.

	DRY (*n* = 13)	WET (*n* = 13)	*p* value
Dry-first arm^∗^	1.92 ± 1.19	1.92 ± 1.25	>0.999
Wet-first arm^∗^	2.08 ± 0.10	1.39 ± 1.20	0.031

DRY: dry technique; WET: wet technique; SD: standard deviation. ^∗^Mean ± SD.

**Table:** (a) Analysis of the specimen cellularity obtained by DRY

Variable factor	Univariate analysis^∗^ *p* value	Multivariate analysis^∗∗^ OR (95% CI), *p* value
Study arm (dry-first arm vs. wet-first arm)	0.322	
Tumor size (<20 vs. ≧20 mm)	0.509	
SEL location (others vs. upper stomach)	0.183	0.514 (0.056–4.697), 0.555
Final diagnosis (GIST vs. others^∗∗∗^)	0.028	9.079 (1.012–81.485), 0.049

**Table:** (b) Analysis of the specimen cellularity obtained by WET

Variable factor	Univariate analysis^∗^ *p* value	Multivariate analysis^∗∗^ OR (95% CI), *p* value
Study arm (dry-first arm vs. wet-first arm)	0.107	4.614 (0.662–32.141), 0.123
Tumor size (<20 vs. ≧20 mm)	0.412	
SEL location (others vs. upper stomach)	0.032	0.125 (0.018–0.858), 0.034
Final diagnosis (GIST vs. others^∗∗∗^)	0.230	

EUS-FNA: endoscopic ultrasound-guided fine-needle aspiration; SEL: subepithelial lesion; GIST: gastrointestinal stromal tumor; DRY: dry technique; WET: wet technique; OR: odds ratio; CI: confidence interval. ^∗^Chi-squared test or Fisher's exact test. ^∗∗^Multivariate logistic regression analysis (acellular and poor cellularity; cellularity scores of 0 and 1 vs. moderate and high cellularity; scores of 2 and 3). ^∗∗∗^Including cases which were not diagnosed by EUS-FNA.

## Data Availability

The data in the paper is all we have, and there is no website that discloses it. However, we will disclose the data if requested by the editor.
